# Met and unmet needs of homeless individuals at different stages of housing reintegration: A mixed-method investigation

**DOI:** 10.1371/journal.pone.0245088

**Published:** 2021-01-14

**Authors:** Marie-Josée Fleury, Guy Grenier, Judith Sabetti, Karine Bertrand, Michèle Clément, Serge Brochu

**Affiliations:** 1 Department of Psychiatry, McGill University, Montreal, Quebec, Canada; 2 Douglas Mental Health University Institute Research Centre, Montreal, Quebec, Canada; 3 McGill University School of Social Work, Montreal, Quebec, Canada; 4 Département des Sciences de la Santé Communautaire, Université de Sherbrooke, Sherbrooke, Quebec, Canada; 5 Département de Médecine Sociale et Préventive, Université Laval, Quebec City, Quebec, Canada; 6 Département de Criminologie, Université de Montréal, Montreal, Canada; Institute of Mental Health, SINGAPORE

## Abstract

This study aimed to identify and compare major areas of met and unmet needs reported by 455 homeless or recently housed individuals recruited from emergency shelters, temporary housing, and permanent housing in Quebec (Canada). Mixed methods, guided by the Maslow framework, were used. Basic needs were the strongest needs category identified, followed by health and social services (an emergent category), and safety; very few participants expressed needs in the higher-order categories of love and belonging, self-esteem, and self-actualization. The only significant differences between the three housing groups occurred in basic needs met, which favored permanent housing residents. Safety was the only category where individuals reported more unmet than met needs. The study results suggested that increased overall access to and continuity of care with family physicians, MD or SUD clinicians and community organizations for social integration should be provided to help better these individuals. Case management, stigma prevention, supported employment programs, peer support and day centers should particularly be more widely implemented as interventions that may promote a higher incidence of met needs in specific needs categories.

## Introduction

Homelessness is an increasing social problem [1]. It is difficult to estimate the real number of individuals experiencing homeless due to lack of participation from many communities in the count, and lack of consistency in methodologies. In 2016, an estimated 550,000 individuals in the US experienced homelessness [1], while in Canada, 235,000 individuals were homeless overall, or 35,000 per night [2]. Compared with the general population, homeless individuals experience more mental health disorders (MHD), substance use disorders (SUD), and acute or chronic physical illnesses (e.g. tuberculosis, human immunodeficiency virus-HIV) [3, 4]. It is estimated that 30–60% of homeless individuals have MHD [5], 50% of whom have co-occurring MHD-SUD [6], while chronic physical illnesses affects about 46% to 85% of this population [7, 8]. Overall life expectancy may be reduced as much as 30 years among homeless individuals as compared with the general population [9].

There is no universal definition of homelessness. According to Busch-Geertsema et al. [10], homelessness may be defined as a lack of access to minimally adequate housing, with adequacy conceptualized along three dimensions: security (e.g. affordability), physical environment (e.g. quality of accommodation), and social conditions (e.g. opportunities for socialization but also privacy). In Canada, homelessness is also defined as a situation in which individuals do not have an adequate, permanent, safe, and stable housing, while lacking the means and the ability to acquire it [11]. Homeless individuals include: 1) those without shelter (e.g. occupants of public spaces, cars); 2) emergency shelter users, and 3) temporary housing occupants in resources provided by community or public organizations, in motels, or with friends [11]. Irrespective of subcategories, homeless individuals share similar challenges in meeting their needs: harsh living conditions [12], food insecurity [13], unemployment [14, 15], poverty [14], social isolation [16], risk of assault [17], and barriers to health care and other services [18–20]. Approximately one third receive minimal treatment for health problems for reasons such as lack of health insurance, transportation problems or discrimination by health professionals [4, 21]. The difficulties experienced by homeless individuals in accessing primary or preventive care may partly explain their high use of emergency departments and hospitalization rates [22], estimated to be 0.5 to 5 times higher than rates for the general population [23]. A US study estimated the unmet health needs of homeless individuals as 6 to 10 times higher than health needs in the general population [20].

Few studies have investigated overall categories of met and unmet needs among homeless individuals [24]. The long-standing Maslow hierarchy of needs [25] remains perhaps the most comprehensive framework for systematic, open-ended investigations of needs in various life domains. Maslow ranged human needs in hierarchical progression from physiological (basic) needs, to safety, love and belonging, self-esteem and, at the peak of the pyramid, self-actualization [26]. According to Maslow, once a particular need is met another gradually takes its place. This last notion has been criticized, however [27–29]. Likewise, studies on homelessness have not always found links between meeting basic needs as opposed to those in other domains [24, 30].

According to research with homeless individuals, housing is the most essential need, followed by the need for regular meals [31, 32] and adequate clothing [31]. Physical needs revolve around access to health care: medical, surgical, and dental services [33], and substance use treatment [31]. Safety needs concern steady income [34], transportation, education [20, 33], and employment [14, 15]. Needs related to love and belonging have concerned lack of supportive relationships with friends and relatives [35, 36], homeless peers [35], and acquaintances developed through shared housing or interactions with landlords, employers, and shelter staff [35]. Studies on homeless youth have identified self-esteem as a protective factor against psychological distress [37, 38]. Among very few studies concerning the need for self-actualization of homeless individuals, two focused on self-actualization among homeless men [39, 40]. One study found that self-actualization may occur while certain needs in other categories remain unmet [24].

Most previous studies have investigated needs among unsheltered individuals or emergency shelter users [33, 39, 40], excluding temporary housing occupants and those who have overcome homelessness such as permanent housing residents. Two opposing approaches exist for moving people from homelessness to permanent housing. The traditional approach, residential treatment first, involves placing individuals in staff-run transitional housing aimed at progressive transition toward permanent, independent housing [41] after they have developed the skills necessary for social integration. By contrast, Housing First offers direct access to permanent housing without requiring other skills, using a harm reduction approach and offering choice in services [42]. These two housing approaches thus differ with respect to the order in which needs should be met. In residential treatment first, health and socialization needs (i.e. love and belonging) must be met before accessing permanent housing, whereas Housing First prioritizes direct access to permanent housing before higher order needs are addressed [24]. Research has assessed resident needs mainly among those living in Housing First. Studies found an association between Housing First and presence of fewer unmet health needs [16, 43, 44]. The impact of temporary housing (i.e. residential treatment first) on resident needs has been less studied [8, 45]. Moreover, to our knowledge, no studies have compared needs among emergency shelter users, temporary housing occupants (i.e. individuals placed in transitional housing based on the resident treatment first approach), and permanent housing residents (similar to the Housing First approach). Only one study [24] has compared temporary housing occupants with permanent housing residents based on the Maslow model. Considering the lack of comparative research, a study that explores needs and challenges across different housing types may help guide decision-making around the reduction of unmet needs through housing interventions or programs.

Using a mixed-method investigation, this study aimed to identify the major areas of met and unmet needs among 455 homeless or recently housed individuals in Quebec (Canada), who represented three distinct types of housing or accommodation: emergency shelter users, temporary housing occupants, and permanent housing residents. Based on Maslow`s theory and previous research [24, 30, 40], we hypothesized that permanent housing residents would identify more met needs or fewer unmet needs than the other two groups.

## Materials and methods

### Study design and data collection

The study was conducted in two urban areas of Quebec with the largest homeless populations and proportions of beds provided in emergency shelters or temporary housing (86% of provincial totals) [46]. Recruitment and data collection in 27 community or public organizations occurred between January and September 2017. Five organizations were emergency shelters, 12 were temporary housing sites, and three permanent housing with supports (similar to the Housing First approach). In Quebec, as mostly elsewhere, emergency shelters offer short-term lodging for people without a roof for the night, meeting their immediate basic needs (e.g. meals, warmth) [11]. Some shelters are dedicated to a specific client types (e.g. women, young people). Temporary housing is a mid-term accommodation (usually three to 24-month duration). Based on a residential treatment approach, temporary housing usually imposes restrictions, such as not consuming alcohol or drugs, and imposes obligations such as participation in individual or collective activities aimed at skill acquisition necessary for autonomous living and social reintegration [47]. Mainly based on the Housing First approach in this study, permanent housing is a long-term accommodation with few obligations for users, and offering a case manager support [42]. In Quebec, both temporary and permanent housing may be offered by public or community organizations. Of the 27 organizations where recruitment took place, seven organizations offered other services such as food banks, employment services, or material support to the homeless population.

Participation in the study was voluntary. Study participants had to be at least 18 years old, with current or previous experience of homelessness and, for those recently housed, be living in permanent housing for less than two years. They were informed of the study through posters displayed in common areas of the selected organizations and postcards. Additionally, meetings were held with organizational staff to present the research and enlist help with recruitment. Moreover, with the authorization and in the presence of the organizational staff, the project coordinator provided short presentations to users on the research during regular activities (e.g. collective meals), following which the names of potentially interested study participants were collected. Interviews took place in the organizations, at individual residences, or in local cafés, usually on the same day or the day following initial contact. Individuals too intoxicated or disorganized to withstand an interview were retained but invited to participate at a later date. Individuals recruited in the seven organizations offering other services for homeless individuals had to provide the name of a resource offering housing, or the telephone number of a professional working in such an organization, who could confirm they used an emergency shelter, or lived in temporary, or permanent housing. Interviews were conducted by research assistants trained and closely monitored by the project coordinator and researchers. Interviewers rigorously followed an interview guide providing detailed information on the participant questionnaire including the open-ended questions, prepared to sustain the interview quality. Questionnaires were also reviewed on a regular basis by the project coordinator to ensure data quality, including that all questions were fully responded. In addition, data analysis was performed several times throughout the interview process to ensure reliability between interviewers. All participants received a modest financial compensation for their time and contribution to the study.

Regarding the interview process, participants completed a questionnaire with both quantitative and qualitative items. The quantitative component included socio-demographic characteristics (age, sex, country of birth, education, employment, marital status); housing history (chronic homelessness—defined as homelessness of at least 12-month duration, or occurring four or more times over the preceding three years [48], reasons for loss of housing); access to health care (e.g. has family physician, use of emergency department); and diagnoses (e.g. MHD, SUD). Standardized instruments used to measure access to health care and diagnoses are presented in the S1 Appendix of the article.

The qualitative component included four open-ended questions, also presented in the S1 Appendix, on: 1) the main difficulties or needs as expressed by the participant; 2) satisfaction with emergency shelters, temporary housing, or permanent housing in response to stated needs; 2.a) whether the responses indicated satisfaction (i.e. met needs) or dissatisfaction (i.e. unmet needs), participants were asked to describe or justify their responses. Individuals were also asked about: 3) how resources other than their housing or accommodations responded to their needs; 3a) if these resources were satisfactory (met need) or not (unmet need), they were asked to describe or justify these responses as well. Finally, participants were asked: 4) how their situation could be improved whether in relation to their housing, other accomodations, or in their overall life to better respond to their needs. According to Perreault et al. [49], open-ended questions are more appropriate than standardized instruments for identifying main sources of satisfaction or dissatisfaction of users with services. Open questions allow participants to express their ideas more directly and freely and to identifying their key priorities in terms of needs than in standardized questionnaires based on a pre-selected categorization of needs. The full interviews took one hour on average to complete, and the qualitative component was audiotaped. Participants provided written informed consent, and the research ethics board of the Douglas Mental Health University Institute approved the multisite study protocol.

### Conceptual framework

**Fig 1:**
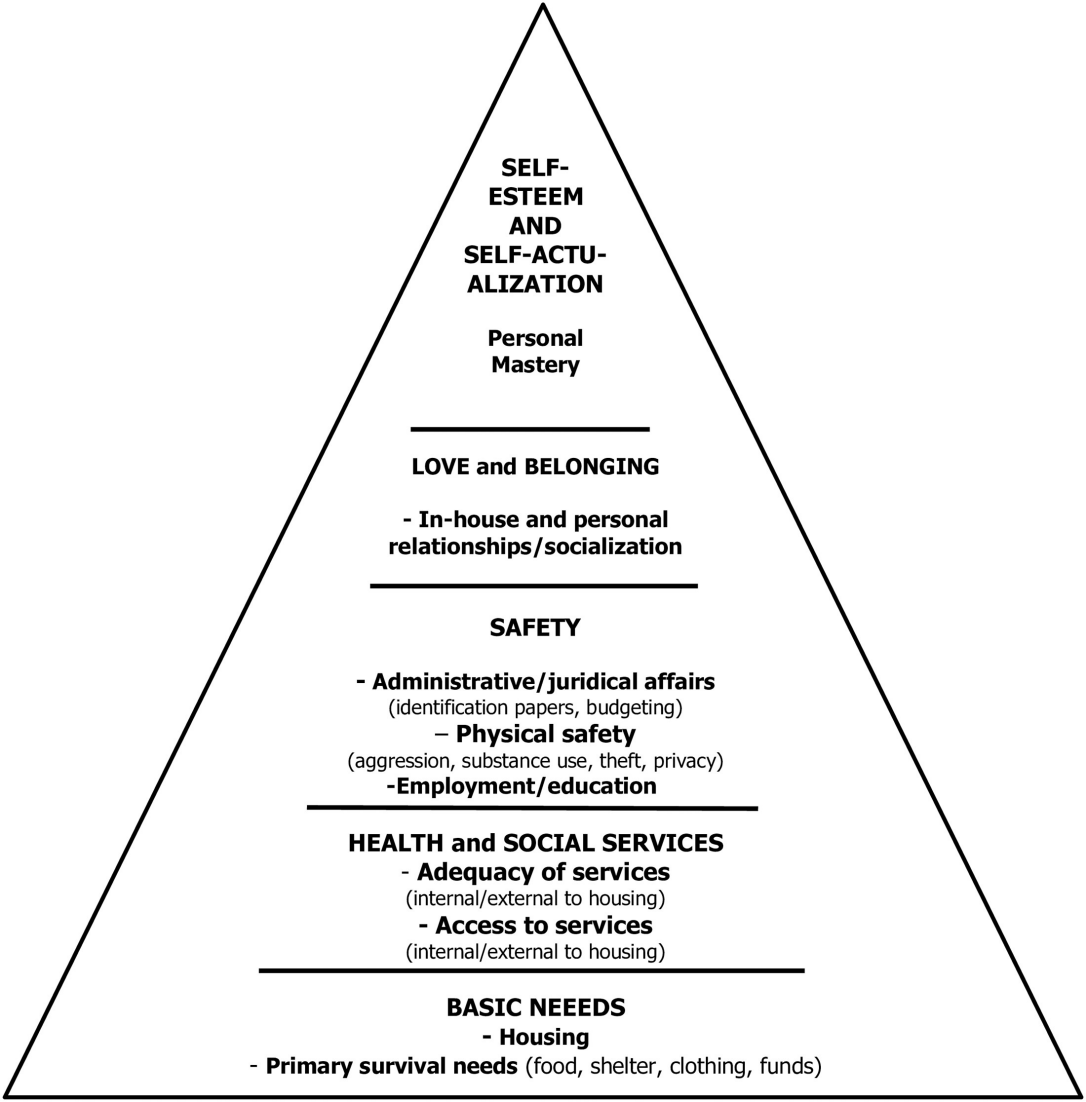
Adaptation of the Maslow hierarchy of needs to a homeless population.

A guiding framework adapted from the Maslow hierarchy of needs was constructed based on five conceptual categories (Fig 1). Basic needs subsumed two sub-categories: primary survival needs (e.g. food, clothing, material support), and housing. An emergent category, health and social services, was developed, with subcategories on access to health services and adequacy of services, given the high prevalence of health needs in this population [19]. The third category, safety, concerned threats to physical safety (e.g. aggression, theft), administrative/juridical affairs (e.g. help with identification papers, health cards, money management) as well as employment or educational needs. Love and belonging, category four, included intimate relationships and friendship [50], as well as socialization within the housing with service providers and peers. As this study found little data on self-esteem and self-actualization, these were defined as a single category, personal mastery, which documented the transformative experience of moving from the streets toward housing and social reintegration. As well, these two categories were usually linked according to perceptions of the few participants who have reported needs in this area.

#### Analysis

The study is based on a mixed method using a sequential explanatory design [51], whereby qualitative findings are converted into quantitative data, and the latter complemented the qualitative information. The quantitative data (i.e. sociodemographic characteristics, housing history, access to health care, diagnoses) were first screened for missing values (which were less than 5%), univariate outliers, and normality assumptions (skewness and kurtosis). Univariate analyses were performed, including number and percentages for the total sample. Comparison analyses were run to assess statistical differences between the three groups (emergency shelter users, temporary housing occupants, and permanent housing residents) for each variable; and integrating the qualitative data converted into the reported needs, between total met vs. unmet needs provided for each category and subcategory of needs across the three housing conditions, using chi-square tests for categorical variables and t-tests for continuous variables.

The framework adapted from the Maslow hierarchy of needs was used for the qualitative and mixed-method analyses following a content analysis procedure. More specifically, data coding and qualitative analysis followed a five-step process: 1) Interviews were transcribed verbatim. 2) Two research team members independently read 10% of the interviews, organized by housing condition, and identified needs based on the Maslow model for the three groups [52] (researchers validated interrater reliability at roughly 90%). 3) Data were separated into met/unmet needs. 4) Met and unmet needs from the qualitative data were than quantified by calculating numbers and percentages of respondents for each category and subcategory of needs, and for met and unmet needs, across the three housing conditions, and comparative analyses produced, as described in the preceding paragraph. 5) Finally, the most important elements of met and unmet needs among participants were described and justified for each category and according to the three housing conditions.

## Results and discussion

### Sample characteristics and group comparisons

In total, 46 emergency shelter users, 242 temporary housing occupants and 208 permanent housing residents (N = 497) were invited to participate in the study, and 455 participated for a 92% response rate. They represented 45/46 (98%) of emergency shelter users, 229/242 (94%) of temporary housing occupants, and 181/208 (87%) of permanent housing residents. While most participants were male, significantly fewer males were in the permanent housing group as compared with temporary housing or emergency shelters. Mean age of the participants was 48 years (SD: 11.2). Most (86%) were born in Canada, with significantly more Canadian-born emergency shelter users than permanent housing residents or temporary housing occupants. Nearly all (94%) were single, significantly more among temporary housing occupants than permanent housing residents. Compared with permanent housing residents, temporary housing occupants reported significantly more SUD or financial problems related to housing loss. Chronic homelessness was experienced by 48%, more among permanent housing residents (56%) than temporary housing occupants (43%) or emergency shelter users (36%). Regarding health care utilization, 51% had a family physician, and 44% a case manager. Significantly more permanent housing residents had a family physician or a case manager than those in the other groups. Emergency shelter users made significantly more use of emergency departments than either permanent housing residents or temporary housing occupants. Permanent housing residents were also significantly less likely to be hospitalized than emergency shelter users and temporary housing occupants. Overall, 72% of participants were diagnosed with MHD, 39% with SUD, and 36% with co-occurring MHD/SUD; 28% had chronic physical illnesses, and 49% acute physical illnesses. Significantly fewer permanent housing residents reported SUD, or overall physical illnesses than did emergency shelter users or temporary housing occupants. Permanent housing residents also had significantly fewer co-occurring MHD-SUD, and overall physical illnesses than temporary housing occupants (Table 1).

**Table 1:** Participant characteristics and comparison between the three groups (n = 455).

			Total sample		Housing categories						Pearson Chi-Square Tests			
					Emergency shelter (N = 45)		Temporary housing (N = 229)		Permanent housing (N = 181)		Total sample	Emergency shelter versus Temporary housing	Emergency shelter versus Permanent housing	Temporary housing versus Permanent housing
Variable categories			n	%	n	%	n	%	n	%	P	P	P	P
Socio-demographics	Age categories	18–39 years	28	6.2	1	2.2	17	7.4	10	5.5	0.348	0.192	0.580	0.343
		40–49 years	194	42.6	17	37.8	104	45.4	73	40.3				
		50 and over	233	51.2	27	60.0	108	47.2	98	54.1				
	Sex	Male	274	60.2	34	75.6	155	67.7	85	47.0	<0.0001	0.297	0.001	<0.0001
		Female	181	39.8	11	24.4	74	32.3	96	53.0				
	Country of birth	Canada	389	85.5	44	97.8	184	80.3	161	89.0	0.002	0.004	0.068	0.018
		Other	66	14.5	1	2.2	45	19.7	20	11.0				
	Education	Secondary or less	310	68.1	36	80.0	159	69.4	115	63.5	0.088	0.153	0.036	0.208
		College or more	145	31.9	9	20.0	70	30.6	66	36.5				
	Marital status	Single	429	94.3	42	93.3	221	96.5	166	91.7	0.111	0.322	0.719	0.036
		Married/common law	26	5.7	3	6.7	8	3.5	15	8.3				
	Has employment		40	8.8	2	4.4	17	7.4	21	11.6	0.185	0.478	0.155	0.147
Housing history	Chronic homelessness		217	47.7	16	35.6	99	43.2	102	56.4	0.007	0.340	0.012	0.008
	3 most frequent reasons for loss of housing/accommodation	Substance use disorders (SUD)	152	33.4	13	28.9	99	43.2	40	22.1	<0.0001	0.074	0.336	<0.0001
		Conflict/violence	141	31.0	15	33.3	73	31.9	53	29.3	0.800	0.848	0.596	0.572
		Financial problems	129	28.4	11	24.4	77	33.6	41	22.7	0.041	0.228	0.798	0.015
Access to healthcare	Has a family physician		193	42.4	16	35.6	79	34.5	98	54.1	<0.0001	0.892	0.026	<0.0001
	Has a case manager		122	26.8	7	15.6	52	22.7	63	34.8	0.005	0.286	0.012	0.007
	Service utilization in past 12 months	Health and social services	349	76.7	39	86.7	174	76.0	136	75.1	0.009	0.003	0.030	0.163
		Emergency department (ED) use	256	56.3	34	75.6	117	51.1	105	58.0	<0.0001	<0.0001	0.003	0.079
		Hospitalizations	173	38.0	29	64.4	72	31.4	72	39.8	0.042	0.049	0.012	0.367
Health status	Diagnosis	Mental health disorders (MHD)	299	65.7	30	66.7	145	63.3	124	68.5	0.541	0.669	0.812	0.272
		SUD	177	38.9	23	51.1	101	44.1	53	29.3	0.002	0.388	0.006	0.002
		Co-occurring MHD/SUD	161	35.4	18	40.0	97	42.4	46	25.4	0.001	0.770	0.052	<0.0001
		Overall physical illnesses	348	76.5	34	75.6	190	83.0	124	68.5	0.003	0.239	0.356	0.001
		Chronic physical illnesses (e.g. hypertension, diabetes)	125	27.5	9	20.0	54	23.6	62	34.3	0.028	0.380	0.045	0.012
		Acute physical illnesses (e.g. impetigo, dental problems)	223	49.0	25	55.6	136	59.4	62	34.3	<0.0001	0.375	0.007	<0.0001

#### Overview of reported needs by category

Table 2 presents summary statistics for the five categories of needs. Basic needs (86%) were most often reported, followed by needs related to health and social services (78%), safety (66%), love and belonging (30%) and self-esteem or self-actualization (20%). Unmet needs were higher than met needs regarding safety (43%), while met and unmet needs were nearly equal for basic needs (59% and 57% respectively). Met needs were higher than unmet needs in the three remaining categories (57% vs 38% for health and social services; 20% vs 11% for love and belonging; 8% vs 2% for self-esteem and self-actualization).

**Table 2:** Summary of perceived met, unmet and total needs within the five conceptual domains of the Maslow hierarchy of needs.

Total individuals with met needs (N = 455)	Total individuals with unmet needs (N = 455)	Grand total needs (individuals)* (n = 455)
**BASIC NEEDS**		
N = 269 (59.1%)	N = 260 (57.1%)	N = 392 (86.2%)
**HEALTH & SOCIAL SERVICES**		
N = 260 (57.1%)	N = 173 (38.0%)	N = 354 (77.8%)
**SAFETY**		
N = 177 (38.9%)	N = 196 (43.1%)	N = 299 (65.7%)
**LOVE & BELONGING**		
N = 90 (20.0%)	N = 52 (11.4%)	N = 132 (29.0%)
**SELF-ESTEEM & SELF-ACTUALIZATION**		
N = 82 (18.0%)	N = 9 (2.0%)	N = 91 (20.0%)

*Since some categories consisted of more than one subcategory (see Table 3), the Grand total needs for these categories does not equal the sum of Total met needs and Total unmet needs. As well, it was possible for the same individual to report more than one met or unmet need across the subcategories, so that the sum of met and unmet needs could exceed 100%. For this reason, we counted one response per person in calculating the Grand total needs for individuals.

#### Percentages of reported needs by category for emergency shelter users, temporary housing occupants and permanent housing residents

Table 3 presents findings for the five categories on total needs and for met and unmet needs across the three housing conditions. Three of the five categories also included sub-categories. Regarding the percentages of participants reporting needs, no significant differences emerged among the three groups. Comparisons of total met versus unmet needs were also similar between emergency shelter users, temporary housing occupants and permanent housing residents. The only significant difference among the three groups concerned total met versus unmet basic needs (*X*^2^ = 7.2; p = .02), where total met needs were higher than unmet needs for permanent housing residents (65% vs. 48%) and for emergency shelter users (60% vs 56%), whereas total unmet needs were higher than met needs for temporary housing occupants (60% vs 56%).

**Table 3:** Perceived met and unmet needs among individuals in three housing conditions based on the five categories in the Maslow hierarchy of needs.

	Emergency Shelters (N = 45)			Temporary Housing (N = 229)			Permanent Housing (N = 181)			P value
	Met needs N (%)	Unmet needs N (%)	Total needs N (%)	Met needs N (%)	Unmet needs N (%)	Total needs N (%)	Met needs N (%)	Unmet needs N (%)	Total needs N (%)	
**BASIC NEEDS**										
**Primary survival needs**	22 (48.9)	9 (20.0)		95 (41.4)	84 (36.7)		86 (47.5)	53 (29.3)		
**Housing**	10 (22.2)	20 (44.4)		56 (24.4)	106 (46.3)		74 (40.9)	60 (33.1)		
**TOTAL INDIVIDUALS**	27* (60.0)	25 (55.5)	38* **(84.4)**	124 (55.9)	149 (65.1)	**202 (88.2)**	118 (65.2)	86 (47.5)	**152 (84.0)**	**0.02**
**HEALTH & SOCIAL SERVICES**										
**Access to services**	4 (8.9)	4 (8.9)		34 (14.8)	44 (19.2)		25 (13.8)	16 (8.8)		
**Adequacy of services**	16 (35.6)	15 (33.3)		139 (60.7)	88 (38.4)		108 (60.0)	57 (31.5)		
**TOTAL INDIVIDUALS**	14 (31.1)	17 (37.8)	**25 (55.6)**	135 (59.0)	92 (40.2)	**185 (80.8)**	111 (60.7)	64 (35.3)	**144 (79.5)**	**0.9**
**SAFETY & SECURITY**										
**Physical safety**	8 (17.8)	9 (20.0)		65 (28.0)	47 (20.5)		39 (21.5)	27 (14.9)		
**Administrative/juridical affairs**	3 (6.7)	8 (17.8)		25 (11.0)	47 (20.5)		23 (12.7)	26 (14.4)		
**Employment/education**	2 (4.4)	8 (17.8)		24 (10.5)	36 (15.7)		17 (9.4)	25 (13.8)		
**TOTAL INDIVIDUALS**	11 (24.4)	18 (40.0)	**24 (53.3)**	88 (38.4)	100 (43.7)	**154 (67.2)**	78 (43.1)	71 (39.2)	**121 (66.8)**	**0.76**
**LOVE & BELONGING**										
**In-house and personal relationships/ socialization**	9 (20.0)	3 (6.7)		53 (23.1)	39 (17.0)		28 (15.5)	13 (7.2)		
**TOTAL INDIVIDUALS**	9 (20.0)	3 (6.7)	**12 (26.7)**	53 (23.1)	36 (17.0)	**81 (35.3)**	28 (15.5)	13 (7.2)	**41 (22.5)**	**0.3**
**SELF-ESTEEM & SELF-ACTUALIZATION**										
**Personal mastery**	11 (24.4)	3 (6.7)		31 (13.5)	3 (1.3)		40 (22.1)	3 (1.7)		
**TOTAL INDIVIDUALS**	11 (24.4)	3 (6.7)	**14 (31.1)**	31 (13.5)	3 (1.3)	**34 (15.0)**	40 (22.1)	3 (1.7)	**43 (23.8)**	**0.2**

* In categories with more than one sub-category, it was possible for the same individual to respond multiple times across the subcategories. In calculating the totals for met and unmet needs, each individual was counted only once. Thus, the totals represent the number of individual responses for each category/housing condition.

#### Participant comments on needs for the three housing groups

Regarding basic needs, unmet primary survival needs mainly described feelings of insecurity around food sufficiency, sleep, and shelter in the short and medium term (see S2 Appendix for illustrative quotations). Some emergency shelter users (about 20%) deplored the dangerous conditions on the streets, limited places in shelters, problems with bed bugs, and the obligation to leave in the morning. Temporary housing occupants expressed less stress related to housing, knowing they were secure for some fixed duration. Yet about half of them felt anxious about their long-term situation, and the lack of available places in permanent housing that caused long wait times. Permanent housing residents expressed a greater sense of stability in terms of their ability to fulfill daily needs and worried less about the future. However, close to 30% of users were concerned about their living conditions, as many housing units needed urgent renovation. As almost all received social assistance as their main source of income, they were often forced to rely on food banks after paying the rent. They most often referred to meet needs for housing (about 65%), whereas the two other groups said more about unmet needs (about 60% and 55% respectively). Most of them enjoyed a sense of freedom in having their own housing despite poor conditions.

Regarding needs for access to health and social services, participants in all groups reported good experiences with providers who offered information and linked them with services. Unmet needs included: problems of access to specialized MH and addiction services or community-based services, and long waits for treatment due to staff shortages, inability to access professional services not covered by health insurance, and lack of individualized MH services in primary care. Few participants (about 10%) also complained about the need to be proactive in seeking help. Emergency shelter users and temporary housing occupants also feared that leaving these accommodations would jeopardize care continuity. Concerning adequacy of health services, all participants expressed appreciation for the availability and listening capacity of most service providers they encountered. Their unmet needs concerned perceptions of some professional incompetence and poor communication or judgemental attitudes. Lack of supervision and frequent turnover were other negative aspects frequently highlighted. Few permanent housing residents (about 10%) greatly resented apartment checks by staff particularly when unannounced, and over-supervision more generally.

As for met safety needs, the sense of physical safety was enhanced for all participants by staff presence and surveillance cameras. However, safety needs were mainly reported as unmet. Unmet physical safety needs concerned instances of theft, violence, and for emergency shelter users and temporary housing occupants, the inability of staff to intervene in crises, which created considerable insecurity. For emergency shelter users and temporary housing occupants, the presence of individuals with MHD or SUD increased fear and insecurity.

Regarding administrative/juridical affairs, unmet needs in all three groups concerned paperwork and other problems dealing with government agencies to obtain documents (e.g. health insurance, social insurance cards). Additionally, very few (less than 5%) participants reported receiving guidance on personal financial management or income tax. Similarly, very few (less than 5%) participants reported met needs in the areas of education or employment. Most faced barriers associated with age, permanent disability, or stigma connected with homelessness. Job security was particularly important for permanent housing residents who needed to maintain an apartment and pay bills. About 15% of the study participants said that they faced disability and age-related challenges that impeded their ability to find or maintain employment.

Less than 10% of participants reported met or unmet needs for love and belonging. For emergency shelter users and temporary housing occupants, met needs were related to the possibility of meeting people whose experiences were like theirs. Peers could be trusted and were sources of support. However, these two groups were adversely affected by their relationships with individuals having MHD and SUD. For permanent housing residents, meeting love and belonging needs often meant having the freedom to choose their friends and meeting their strong desire for privacy. This led some to prefer isolating themselves and avoiding contact with neighbors.

Finally, only 20% of study participants addressed their self-esteem and self-actualization needs, pointing out opportunities throughout their homeless trajectory to build skills such as autonomy, optimism, and resilience. Despite relying on help from various sources, many of these participants acknowledged their personal responsibility for improving their lives. In terms of met needs, permanent housing residents expressed mainly sense of accomplishment, lessons learned, and overall satisfaction at having gained some independence, whereas emergency shelter users and temporary housing occupants tended to be more prescriptive, or wishful about developing skills.

Overall, this study identified and compared perceived needs among homeless (emergency shelter users, temporary housing occupants) and recently housed individuals (permanent housing residents) in Quebec, using an adapted version of Maslow hierarchy of needs. Findings validated the relevance of this model for research on homelessness, with met/unmet basic needs most often reported and decreasing numbers of needs identified in ascending the pyramid. Significant group differences emerged for basic needs only, with more met needs among permanent housing residents versus the other two groups. This indicates that, for homeless individuals, meeting basic needs, including housing, was not a sufficient condition for meeting needs in the higher categories. It also underlined that meeting basic needs was not necessary to develop and potentially meet needs in the higher categories, such as health and social services or love and belonging, which confirmed previous criticism of the original Maslow framework [27–29].

#### Comparison with other study samples

Our sample characteristics reflect differences as well as similarities with samples observed in previous research. The main difference concerned the very high proportion of participants with a history of chronic homelessness in this study, affecting almost half of the sample. This may be explained by the fact that the great majority of participants in this study were temporary housing occupants or permanent housing residents, which would be logical as these programs were geared to chronically homeless individuals [41], and included relatively few emergency shelter users, as opposed to the samples in most previous studies. For example, only 10% of the US homeless populations is estimated to experience chronic homelessness [53]. The over-representation of chronically homeless participants in our study, particularly among permanent housing residents, might also explain the high prevalence of participant health problems [53]. In terms of health and social service use, our sample had the high rates of service utilization typical for homeless populations [4, 54, 55]. The three groups reported twice as much use of emergency departments over a 12-month period relative to the general population, and had more hospitalizations, particularly emergency shelter users whose hospitalization rates were nearly four times the norm for the general population as shown in previous studies [23, 56].

#### Sample characteristics and group comparisons

Statistical comparisons among the three housing categories (Table 1) revealed that permanent housing residents enjoyed significant advantages (e.g. more with a family physician and case manager support, lower SUD and co-occurring MHD/SUD rates) as compared with other groups. This supported our hypothesis that permanent housing residents would have relatively more met, or fewer unmet, needs than other groups. Research indicates that having family physicians and case managers likely facilitated residential stability among homeless individuals [57] allowing them to make less use of emergency departments and reducing hospitalizations [44, 55]. The low proportion of permanent housing residents with a case manager seems however to indicate that most did not live in permanent housing with support, as recommended in the Housing First approach. This may partly explain the persistence of several unmet needs in this group. Moreover, the fact that SUD was the main reason for housing loss among temporary housing occupants seems to indicate that homeless individuals with SUD may be viewed as less favorably by permanent housing programs. The original Pathway to Housing model focused on homeless individuals with MHD or co-occurring MH/SUD [58], yet the Housing First approach has since expanded to include other types of clients, and may also include a diversity of organizational features that were not part of the original model. Those with SUD exclusively may have been relegated to other residential settings. Moreover, the greater prevalence of SUD, physical illnesses and co-occurring MHD/SUD among temporary housing occupants and emergency shelter users might explain their greater use of emergency departments and more frequent hospitalizations than among permanent housing residents.

The qualitative findings also reflected a distinction between permanent housing residents and others in terms of the greater autonomy and the better level of comfort offered by permanent housing. This coincides with findings in Henwood et al. [59] suggesting that quality of life among chronically homeless individuals would increase mainly in areas related to living situation following transfer to permanent housing. Yet for emergency shelter users and temporary housing occupants, the lack of housing stability ensured a more precarious situation in terms of meeting basic needs.

#### Group comparisons concerning needs categories

Safety was the only category in which individuals reported more unmet than met needs due to the prevalence of aggression, particularly for emergency shelter users and temporary housing occupants, as well as the administrative problems and unemployment reported by all participants. These factors, already mentioned in previous studies [15, 21, 60, 61], have represented key barriers to effective social integration among homeless individuals, including those living in permanent housing. Aggression has been reported as an important risk factor, suggesting that homeless individuals are particularly victimized by stigma and discrimination from the general population. The verbatim further indicated that emergency shelter users and temporary housing occupants with no MHD or SUD also felt insecure vis-à-vis their counterparts affected by these disorders. Stigma was identified in studies [62] as being higher among homeless individuals with MHD and/or SUD then among those in other populations, which may explain why a substantial proportion of permanent housing residents had experienced chronic homelessness and were affected by MHD. The prevalence of administrative problems in the sample may reflect a lack of collaboration among health and social services and homeless services, as evidenced in previous reviews on barriers to care for homeless individuals [21]. Moreover, the high unemployment rate for these study participants who lived mainly on social assistance as their only source of income suggested ongoing insecurity in relation to their ability to cope with unexpected expenses.

The qualitative data also allowed us to identify other interesting distinctions among the groups for the other need categories, even in the absence of significant quantitative differences. Concerning health and social service needs, results show that the lack of access to specialized MH and SUD services as well as to primary care and community-based services was deplored by all groups. However, the responses of permanent housing residents and temporary housing occupants indicated that referrals to appropriate resources by staff may have promoted better adequacy of services, a possibility not available to the shelter group.

Concerning love and belonging, the capacity to meet needs seemed related to participants’ sense of safety in forming relationships. Emergency shelter users and temporary housing occupants reported constant support from individuals with similar problems, which was a positive factor. A US study found that individuals with greater social support have fewer homeless episodes [63]. Yet, having to share close quarters with individuals affected by complex MHD and/or SUD may have undermined the social benefits of group living for many. By contrast and as reported previously [64], some permanent housing residents seemed to avoid relationships that stood to threaten their sobriety or emotional stability. As reported elsewhere [64, 65], needs among permanent housing residents for love and belonging may be overshadowed by desires for autonomy, selectivity in relationships, and privacy. Isolation and loneliness occurring among permanent housing residents, also highlighted in other studies [16, 66], may have been a necessary strategy for ensuring personal security. As a Canadian study reported for women in particular, housing does not solve homelessness unless accompanied by a sense of security [67]. Previous findings have also suggested that permanent housing does not increase social integration [41, 59, 68]; permanent housing residents may avoid contact with their neighbors or social activities, which justifies the importance of building a social network.

Very few study participants expressed met or unmet needs in terms of self-esteem and self-actualization. According to Wenzel et al. [30], these needs would tend to decrease over time among permanent housing residents. By contrast, study findings suggested that emergency shelter users and temporary housing occupants focused more on acquiring needed skills, for example returning to school or engaging in artistic activities. Many participants also took pride in finding the inner strength to survive homelessness, as confirmed previously [39]. Despite only subtle quantitative differences among the three groups, we found anecdotal evidence to suggest that some homeless individuals had the capacity to define their life purpose and a philosophy of life, achieving personal satisfaction despite severe material deprivation, which is similar to results in previous studies [39, 40]. It would however be necessary to conduct further studies, including those using standardized instruments before drawing firm conclusions.

*Clinical and public health implications*. In view of the study results, certain interventions may be recommended for meeting important needs. Concerning health and social services, studies have suggested that case management facilitates access to services and increases continuity of care for homeless individuals [69]. As such, permanent housing “with support” based on the Housing First approach should be more widely implemented into permanent housing resources, and individual case managers assigned, particularly after initial transfer to permanent housing in order to prevent the risk of relapse. Case management should be also more consistently implemented in temporary housing, while improved outreach programs might facilitate access to health and social services among emergency shelter users. Considering the great prevalence of SUD and co-occurring MHD/SUD, especially among emergency shelter users and temporary housing occupants, interventions should be considered that may facilitate referral to addiction rehabilitation centers, including the deployment of addiction liaison nurses in emergency departments. One program closely related to Housing First may be implemented for the treatment and follow-up of homeless individuals affected primarily by SUD, particularly chronic cases. Moreover, greater access to primary care through family physicians would facilitate treatment of physical illnesses, prevalent in all three groups but mainly among temporary housing occupants. Concerning safety needs, stigma prevention for homeless individuals should be promoted. Furthermore, supported employment programs targeted to permanent housing residents may help maintain housing stability [58]. Regarding love and belonging, self-help groups and peer support [70] may contribute to the development of social networks, mainly among permanent housing residents affected by isolation and loneliness. Finally, a more recovery-oriented system may contribute to an increased sense of personal mastery among homeless individuals [24]. Day centers, for instance, promote various artistic or recreational activities that enhance, through the acquisition of skills, personal autonomy, self-esteem, and realization of a more meaningful life.

### Limitations

This study had some noteworthy limitations. First, since participation was voluntary and non-random, our findings require further validation. Second, emergency shelter users who constitute the bulk of the homeless populations were underrepresented in our study, whereas chronically homeless participants were overrepresented. Thus, the opinions of study participants did not reflect those of all homeless individuals. Third, our sample was recruited exclusively in urban areas. Their needs may be not representative of those from rural or remote areas. Fourth, the findings concerning permanent housing residents may not perfectly coincide with those identified in Housing First studies, as in this study many individuals in permanent housing lacked a case manager. Fifth, the use of open-ended questions to elicit met/unmet needs did not guarantee the inclusion of all possible needs or that the importance of each need was accurately estimated; rather, responses may have reflected more the needs and priorities of the study participants. For example, needs related to love or self-actualization may not have been expressed in the face of more urgent survival needs. As individual needs assessment was not based on a standardized questionnaire, but rather on open-ended questions, the study results needs to be replicated. Finally, this study was cross-sectional. A longitudinal study would have allowed us to observe changes in met and unmet needs across the various categories over time.

## Conclusion

This study makes an original contribution to the literature on needs assessment in homelessness, as the first study to our knowledge to assess met and unmet needs among homeless and recently housed individuals recruited from three types of housing services, and using the Maslow framework in a mixed-method investigation. According to the results, the only significant differences between the three housing groups occurred in the high incidence of met basic needs, favoring permanent housing residents, as hypothesized in this study. However, fulfilling basic needs was not sufficient to increase levels of met needs in the higher categories of the Maslow pyramid. Safety issues, in particular, posed a major barrier to real social integration among homeless individuals, including those recently integrated into permanent housing. Several interventions (e.g. case management, stigma prevention, supported employment program, peer support, day centers), and increased overall access to and continuity of care with family physicians, MD or SUD clinicians and community organizations for social integration may help more individuals satisfy their needs within specific needs categories.

## Supporting information

S1 Appendix Standardized instruments used to measure access to health care and diagnoses; and the qualitative component including four open-ended questions and sub-questions. (DOCX)

S2 Appendix Illustrative quotations on met and unmet needs for the three housing groups. (DOCX)
