# Supplementary material for: Met and unmet needs of homeless individuals at different stages of housing reintegration: A mixed-method investigation
Source: PLoS One. 2021 Jan 14;16(1):e0245088. doi: 10.1371/journal.pone.0245088 (PMC7808646; doi:10.1371/journal.pone.0245088)
Supplement: S1 Appendix — (DOCX) [file pone.0245088.s001.docx]

**S1 Appendix. Standardized instruments used to measure access to health care and diagnoses; and qualitative component included four open-ended questions and sub-questions.**

| **QUANTITATIVE data: Instruments and references** | | | |
| --- | --- | --- | --- |
|  | | | |
| **Variables** | **Instruments & Reference** | **Description** | **Psychometric Properties** |
| Socio-demographic variables | | | |
| Housing status | Canadian Community Health Survey (CCHS)^1^ | Self-report; three types of housing (emergency shelter, temporary housing, permanent housing) | N/A |
| Age |  | Self-report; numerical value calculated from date of birth | N/A |
| Sex |  | Self-report; 2-point scale  (male=1, female=2) | N/A |
| Country of birth |  | Self-report; 2-point scale  (Canada-born=0; foreign-born=1) | N/A |
| Education |  | Self-report; 2-point scale (high school or less=1, college or more=2) | N/A |
| Marital status |  | Self-report; 2-point scale (single/divorced/ widowed and living alone=0; married and living as a couple=1) | N/A |
| Has employment |  | Self-report; two-point scale  (no=0; yes=1) | N/A |
| Housing history | | | |
| Chronic homelessness | Canadian Community Health Survey (CCHS)^1^ | Homeless episodes and duration as declared by participants. Being homeless during at least 12 months or 4 episodes within a 3-year period=chronic homelessness | N/A |
| The three most frequent reasons for loss  of housing/  accommodation |  | 12 choices | N/A |
| Access to healthcare | | | |
| Has a family physician | Service Utilization Questionnaire (SUQ) adapted from CCHS^2^ | Self-report; two-point scale  (no=0; yes=1) | N/A |
| Has a case manager |  | Self-report; two-point scale  (no=0; yes=1) | N/A |
| Frequency of health and social service use |  | Self-report; numerical value based on previous 12 months | N/A |
| Frequency of  emergency department visits |  | Self-report; numerical value based on previous 12 months | N/A |
| Frequency of hospitalizations |  | Self-report; numerical value based on previous 12 months | N/A |
| Health status | | | |
| Diagnoses | | | |
| Mental health disorders (MHD) | M.I.N.I International Neuropsychiatric Interview 6.0^3^ | 120-item structured diagnostic interview for DSM-IV and ICD-10 psychiatric disorders; two-point scale (no=0; yes=1) | Kappa Cohen= 0.50-0.84 |
| Substance use disorders (SUD) | Alcohol Use Disorders Identification Test (AUDIT)^4^ | 10 items (0 to 4 for each variable);  8 and +=hazardous or harmful alcohol use | Cronbach’s alpha=0.68 |
|  | Drug Abuse Screening Test-20 (DAST)^5^ | 20 items (0 to 1 for each variable);  6 +=likelihood of substance use disorders (SUD) | Cronbach’s alpha=0.88 |
| Physical illnesses | Canadian Community Health Survey (CCHS)^1^ | Number of physical illnesses as declared by participants | N/A |

1. Statistics Canada. Canadian Community Health Survey (CCHS). Ottawa: Statistics Canada; 2002.

2. Gravel R, Beland Y. The Canadian Community Health Survey: mental health and well-being. Can J Psychiatry. 2005; 50(10): 573-9.

3. Sheehan DV, Lecrubier Y, Sheehan KH, et al. The Mini-International Neuropsychiatric Interview (M.I.N.I.): the development and validation of a structured diagnostic psychiatric interview for DSM-IV and ICD-10. The Journal of clinical psychiatry. 1998; 59 Suppl 20: 22-33;quiz 4-57.

4. Bohn MJ, Babor TF, Kranzler HR. The Alcohol Use Disorders Identification Test (AUDIT): validation of a screening instrument for use in medical settings. J Stud Alcohol Drugs. 1995; 56(4): 423-32.

5. Skinner HA. The drug abuse screening test. Addict Behav. 1982; 7(4): 363-71.

**QUALITATIVE DATA: OPEN QUESTIONS**

*I will now ask open questions, so I can better understand your needs and your satisfaction with the support you have received in response to your needs or difficulties.*

1. What are your main difficulties or needs?
2. Does your accommodation or housing (i.e. emergency shelters, temporary housing, or permanent housing) respond to your needs?

- If yes, please describe or justify your response (i.e. met needs).
- If no, please describe or justify your response (i.e. unmet needs).

1. Other than the support that you are receiving through your accommodation or housing itself (i.e. emergency shelters, temporary housing, and permanent housing), that can be provided from any other resources, do you find that you receive enough support to response to your needs?

- If yes, please describe or justify your response (i.e. met needs).
- If no, please describe or justify your response (i.e. unmet needs).

1. Overall, what can be improved to support you better whether in your accommodation or housing, or in your overall life more generally?
