# Supplementary material for: Met and unmet needs of homeless individuals at different stages of housing reintegration: A mixed-method investigation
Source: PLoS One. 2021 Jan 14;16(1):e0245088. doi: 10.1371/journal.pone.0245088 (PMC7808646; doi:10.1371/journal.pone.0245088)
Supplement: S2 Appendix — (DOCX) [file pone.0245088.s002.docx]

**S2 Appendix. Illustrative quotations on met and unmet needs in the three housing groups**

| **BASIC NEEDS** | | | | **Emergency shelter users** | | | **Temporary housing residents** | **Permanent housing residents** |
| --- | --- | --- | --- | --- | --- | --- | --- | --- |
| **Primary**  **Survival**  **Needs** | met needs | | | *“It`s like a home here… they gave me a bed, some soap to wash up… to know that I can sleep well in the warmth, and that I’m going to eat. Whatever else (happens), there’s something to eat.”[ESU 1190490]* | | | *“I’ve found a place to sleep… less worry for a while, and that gives some respite. We are well fed, three meals a day and snacks. They are on time with the meals.” [THR 11153461]* | *“…you are alone here. You are free. It isn`t like in temporary housing . . . where there is little privacy and you feel uneasy.”[PHR 11177675]*  *“…they just finished doing renovations in the building. They invested a lot of money to meet peoples’ primary needs.”[PHR 111173722]* |
|  | unmet  needs | | | *“Conditions on the street are dangerous; I lost my toenails from hypothermia. You have to leave the shelter during the day, and find another place.” [ESU 11143556]*  *“The first problem is that there aren`t enough places in the shelters. We need more, because often you arrive at the door, and they are full.” [ESU 111910598]* | | | *“We can only stay here 2 months… so if I don`t find something, I’ll be back in the street.” [THR 11111246]*  *“Cleanliness is a big problem… bedbugs, filth. I sleep fully dressed, just to give you an idea. So I won`t be attacked . . . people arrive at 10 or 11 p.m. to sleep in our dorms; but they aren`t washed, whereas those of us in the program are required to wash every day… there aren`t enough beds, which doesn`t help.” [THR 11144317]”* | *“It’s still heard, even if you have housing… The rent isn`t expensive because you’re on social assistance, but you don`t have much. I have to go to food banks.” [PHR 11173705]*  *“I need to stay on welfare for the security. But I need more than $600 a month. I can’t manage; I’m living on a tightrope and drowning because the government cut my social assistance to pay for my medications.” [PHR 11170580]* |
| **Housing** | met needs | | | *“They help you to find an apartment, yeah.” [ESU 11190589]*  *“Toward Léger Street, there’s a place; it`s not a crack house; there`s some pot, but everyone there is nice… most of the time.”[ESU 11190565]*  *“They (workers) helped me to find a place that specializes in people with hepatitis C, or HIV. Everything is provided. Listen: you have a tv in your room, food provided, complete. It’s really, really fun, but it costs 75% of your revenue.” [ESU 11190598]* | | | *“So I found the right place for me. But people don`t know what the right place is when you live on the street… what is the right fit. At the beginning you gonna tell any lies, anything, just so you can stay inside.” [THR 111110243]* | *“It makes me safe… I have my key and a home, after six years in the street. In temporary housing, you have no choice but to respect the rules… But here, I do what I want, when I want.” [PHR 11173722]*  *“I feel less powerless… having a room gives you the sense of having at least a minimum.” [PHR 11163643]*  *“…You are alone here. You are free. You can cook, do your errands, washing, self-care. You can come back any time you want, go out, and invite friends over. We can do sewing, drawing; you have your own things” [PHR 11177675]*  *“The workers were always after me. I found this hard at first, but it was this kick in the pants that got me my apartment.” [PHR 22299440]* |
|  | Unmet needs | | | *“I have bad credit, which restricts my options…. the landlords are full of prejudice against those who have bad credit… What needs to be improved is that landlords need to stop labelling everyone from the start.” [ESU 11176586]*  *“There should be more places for women. I’ve been waiting for an HLM (social housing) for 15 years, but never got anything.” [ESU 11190592]*  *“Okay, but to get an apartment, you need bus tickets so you can visit places. And you need to buy the newspaper.”*  *[ESU 11190657]* | | | *“Cockroaches. Yeah, so that’s why I wanna move. Only it`s really hard to find something affordable. That’s the only apartment I can afford right now.” [THR 11130312]*  *“For sure, I will need follow-up when I get an apartment; someone who can explain what to do. Because when you start to live in an apartment, it’s your parents who explain things. I didn`t have parents, so I’m a bit lost about all that.” [THR 11121241]*  *“It`s too difficult to get a subsidized apartment. They usually prioritize people without a place, mostly women, single mothers. It’s discouraging to put in a request and wait 1 to 5 years for a response.” [THR 11157557]* | *“The places are not renovated; they’re old. I have water leaking in from the door of my balcony. I’ve been complaining for three years, but they haven’t fixed it yet.” [PHR 11110276]*  *“We need to face reality also… I no longer work, so my revenue is what the government gives. And that sets the limits. I’m going into a coop that fits my budget.” [PHR 11152648]*  *“I asked for a place at the HLM (social housing). If I get it, I won`t stay (here). The wait lists are long, because it`s less expensive. But I would feel like I’m living in my own home. Not like here, where I’m living in my landlord’s place…” [PHR 11177680]* |
| **HEALTH & SOCIAL SERVICES** | | | | | | | | |
| **Access to health services** | | Met needs | | | | *“They gave me a card that asked for access to the addiction center.” [ESU 11190589]*  *“Now they are more understanding. They see the problems people have, and they got a couple of real workers – the others weren’t really professional. So it’s better than before. You have more access to help.” [ESU 11190372]* | *“You can see a psychologist, psychiatrist, all that. I haven`t been here long, nearly two weeks, but I find that we have all the help needed.” [THR 11148247]*  *“What helped me the most was that they pushed me toward the addiction center… They directed me toward services.” [THR 11131553]* | *“The fact of having access to different health and social services (was helpful). The services were attainable.” [PHR 11199720]*  *“I think there is enough information out there that you can find out about health services and the system.” [PHR 11168666]*  *“They (staff) direct me to medical services, and offer moral support. They help me stay stable… When I need to talk, I call the workers. They are my friends. I’ve been with them for 10 years.” [PHR 11164600]* |
|  |  | Unmet needs | | | | *“I’m not covered by insurance for medical treatment. If you have money, you can pay a chiropractor but that costs something like $200 or $300 a week, or something like that.” [ESU 11190570]*  *“I was supposed to get another appointment with the doctor, probably to start treatment. No news yet. I’m waiting. It’s always a bit long with these things.” [ESU 11190598]* | *“There aren’t enough social workers, psychiatrists, psychologists. We get help, but from younger workers just out of school; they never lived in the streets and don`t understand anything. We need proper follow-up, starting with those who most need help. “[THR 11114437]*  *“It isn`t easy to get information (about services). You have to ask constantly to know if there is anything else available.” [THR 11113652]*  *“(We need) follow-up, here but also in the community once we leave here… yes, continuity…” [THR11148253]* | *“ “They told me at the CLSC (local health center) that they don’t offer mental health services any more, due to lack of time. They only have groups. A group might break your emotional solitude, but sometimes it`s just that they don`t want to listen; there is also incompetence. And with the heavy bureaucracy, I can understand why some (professionals) leave the system.” [PHR 111168666]*  *“Nothing helps me. But if I don`t look for something, nobody is going to give me help.” [PHR 11168562]* |
| **Adequacy of services** | | Met needs | | | | *“There are many service providers working here, but only one that listens and tries to help you with your papers and with your needs. They’re not always met, they’re not always accommodated, but you have a sense of hope that somebody is taking care of something. Sometimes that’s enough not to get frustrated.”[ESU 11121391]*  *“The workers… They know how to answer my questions; they know; they have been there.” [ESU 11190565]*  *“They know me at the hospital; they are good, the nurse who works there is a ray of sunshine. I very, very, very well cared for.” [ESU 11190568]* | *“What helps me the most is the support, being accompanied. When things aren`t going well, someone is always there for us.” [THR 11111226]*  *“My worker is professional, but very human at the same time. You feel that she is happy in what she does, as if it weren’t a job. That makes all the difference.” [THR 11135324]*  *“The workers really listen… they don’t judge us.” [THR 11153452]* | *“It’s hard at first… They are welcoming, always listening. They propose solutions that fit your needs, and offer you tools that are really personalized to your needs.” [PHR 22269428]*  *“Their availability – staff, students, volunteers – is the great strength of this house.” [PHR 11174544]* |
|  |  | Unmet needs | | | | *“There’s a great lack of supervision; everything is lax. Nobody offered me any kind of social integration… Everyone has different needs that should be taken into account.” [ESU 11190376]*  *“Some workers are prejudiced.” [ESU 11117250]*  *“Sometimes there is a lack of communication, and judgment.” [ESU 11117250]* | *“I had confidence in one worker who is no longer here. It’s hard to start over with a new person; I really don`t want to… “[THR 11157557]*  *“The staff aren`t professional… some don`t have the competence; it depends on the person.” [THR 11144394]*  *“They say they can help me; I need help; but they are too busy to sit down and talk to me.” [THR 11142446])* |  |
| **SAFETY & SECURITY** | | | | | | | | |
| **Physical safety** | | | Met needs | | *“A place to put my head, and feel safe.” [ESU 11190589]*  *“You can get permission to stay in bed for the day if you are sick, but this is a special permission…” [ESU 11190567]*  *“In the past few years, things have improved a lot, it is more open… Put a camera, and you solve the problem. They finally did that. You know, small improvements…” [ESU 11190598]*  *“There are cameras, except in two or three areas…” [ESU 11190490]* | | *“There was a guy who was violent, and they kicked him out. So they did what they had to… I was happy about that.” [THR 11131553]*  *“Here, you don’t feel afraid that someone is going to come in and make problems. You are protected.” [THR11131354]* | *“I’m not afraid that someone will come in and attack us, or whatever. I feel secure.” [PHR 22299419]*  *“I find that the place is quiet enough. I have my little home, which I’ve set up well. What I like is that it`s super-secure—surveillance cameras, everything. I feel safe here.” [PHR 11110280***]** |
|  |  |  | Unmet needs | | *“Someone cut my locks and stole my personal things, my tax returns, my bank book… It’s identity theft…” [ESU 1119059]*  *“They should have different places for people in mental health, and the addicts, because they are mixed in with the more vulnerable ones… So you’re stuck with one guy who talks to himself, and another high on cocaine, then another who is aching all over…” [ESU 11190592]* | | *“Two people in residence might argue and end up fighting. I don’t react that way. I have a way of expressing what I feel verbally, unlike others who are only able to talk with their fists.” [THR 1114431]*  *“You can’t let people in (housing) programs consume. There have to be sanctions. They let a lot get by…” [THR 11113344]* | *“There’s no staff here on the weekend, but I have to accept it. Security is my big problem because of what I’ve lived through…. We need either a guard or a worker on duty, one or the other…” [PHR 11110275]* |
| **Adminis-**  **trative/**  **judicial**  **affairs** | | | Met needs | | *“They are helping me with my application . . . today it`s just paper, paper, paper . . .” [ESU 11190490]* | | *“…I like to manage my money myself. So I said to her: ‘You can take money for the rent, that doesn’t bother me; at least I have a roof over my head, and I can eat; But the rest, you can give me right away… She said okay.” [THR 11135447]* | *“They offered me housing on condition that I accept curatorship for a year, and I had 24 hours to decide. I found that revolting, but in the end I accepted…” [PHR 11110277]* |
|  |  |  | Unmet needs | | *“…you don`t want to break your head with the landlord. It would help me if they withdrew my rent from my social assistance cheque, and sent me the balance, because we (homeless) are not very well organized. You don`t want to spend all your money, and the landlord shows up 5 minutes later (looking for the rent).” [ESU 11190490]* | | *“They cut $200 from people’s welfare cheques when they are in TH… okay, I agree with that because the person is housed and fed. However, they should start to give it back before people leave there so they will have some money put aside. I don`t know anyone who has left there feeling at ease; it’s a serious risk for relapse.”*  *[THR 11174544]* | *“The big problem I have, where I really need help, or can’t manage on my own, is how to fill out these darn forms that we get. I’m desperate, I don’t understand why I have to fill out so many questions… There are pages of information. This is too much for me; I’m not capable.” [PHR 11171676]* |
| **Employ-ment/**  **education** | | | Met needs | | *“We are people who walk a lot, those of us who are ambitious. I have walked for hours and hours to get to an interview because I didn’t have a cent in my pockets.” [ESU 11142388]* | | *“…you can`t do much with Emploi-Quebec. But, here (TH), if you don`t have any activity, you are obliged to do tasks. It’s like work, even if you aren`t paid; but at least it helps psychologically.” [THR 11121287]* | *“Outside of housing, my job helps me most. I make breakfasts. I have an hour and 15 minutes of work every morning on the toaster.” [PHR 11168548]*  *“I deliver the Itinerary (newspaper) when I’m able.” [PHR 11172678]* |
|  |  |  | Unmet needs | | *“There are some that are made for school then there are others not. Today, it’s paper, paper, paper…” [ESU 11190490]*  *“As for professional training or school. Yes, that helps you afterward to open other doors. Even if it would be just to pass a broom – I did that for three months. A cooking course would help a lot of people.” [ESU 11190549]*  *“An employment service would be good. I need to register for that, but I need to be ready. I have to redo my CV, but I need to go about it gradually, not right away.” [ESU 11190372]* | | *“Employers don’t want us. If you give a shelter as your address, they look at you like you have a MHD, as if you’re on drugs or alcohol… There is no place for a woman who has been unfortunate, who has fallen in a hole and is trying to get herself out at age 60…” [THR 11133230].*  *“We need further assistance in finding employment.”*  *[THR 1131368 ]* | *“I am considered permanently unable to work because my doctor at the time gave me that designation so that I wouldn’t suffer too much financially… but this blocks me from employment programs.” [PHR 11173597]*  *“It’s hard, because I’m 55 years old. I look for work under the table, and survive because that gives me $440 a month.” [PHR 11199375]* |
| **LOVE & BELONGING** | | | | | | | | |
| **Relation-**  **ships/ socializa-**  **tion** | | Met needs | | | | *“What I appreciate the most is to have lots of people together, all ages; to be able to talk, to see their ‘baggage’; to be able to help people with the same stories as me…” [ESU 11190549]*  *“…They saved my life. They accepted me in their place. Respect… They respected me.” [ESU 11190568]* | *“I surround myself with people who are positive. That helps me a lot, and restores my confidence.” [THR 11131539]*  *“I know all the children (in my family) because you can do everything with the Internet. I stay up to date on what they are doing; I know what universities they are attending, and the like. It’s marvellous.” [THR 11141239]*  *“I’m building a new circle of friends who don`t consume, and who are positive.” [THR 11140374]*  *“I have worked a lot on myself to have a better life with my children, and my network – to have a new social network, which has helped me a lot. At one time I would not have been able to sit and talk with you.” [THR 11133224]* | *“We respect each other on the floor; it teaches you to live with people… When I want to be by myself, I’m by myself. So we’ve got the best of both worlds.”*  *[PHR 1110272]*  *“What helps, in general, is that I don’t have close contact with my neighbors, like in my old lodging. They don`t harass me or meddle in my affairs. So I can enjoy the quiet. [PHR 11130313]* |
|  |  | Unmet needs | | | | *“They should have different places for people with MHD and the addicts, because they are mixed in with the more vulnerable ones… So you’re stuck with one guy who talks to himself, and another high on cocaine, then another who is aching all over…” [ESU 11190592]*  *“… Friends… well, often you can`t trust anyone.”*  *[ESU 11190565]*  *“…When I get to the shelter, I don`t eat right away; I watch tv or something, because I’m used to eating with others. When you eat out, people are around for company.”*  *[ESU 11190587]*  *“In terms of not having family or visitors, it wasn’t, like, my place at all… I wanted my family to come around.” [ESU 11190315]* | *“I don`t like to be around people who can`t get along with others. Being able to get along would help stabilize me. But we are all so different. People do the best they can, with who they are, to get along.” [THR 11113651]*  *“If you meet a friend, you go to their place to watch a movie… but you can`t invite them to your place because you don`t want to disclose that you’re living in a homeless shelter. So I find it very embarrassing and humiliating.” [THR 11144273]*  *“Just the communal living with other people who have problems, adds to the problems you have… (e.g. mental health problems.” [THR 11130311]* | *“I have conflicts with half the tenants in my co-op. [PHR 1117058]*  *“They wanted to get people off the streets; so they brought in some who are “shooting up” all day, full of problems. There’s one that has to be kicked out before she messes up everyone on the block… you understand, because not everyone is so nice all the time. “ [PHR 1199715]*  *“People often isolate themselves in their rooms, or in bed. Rather than go, I don`t know, to meetings or whatever, the staff should follow them up.” [PHR 11177738]*  *“I wish I had a worker who would ask if I wanted to go bicycling with her.” [PHR 11164363]*  *“I don’t often go (to house activities) because I like to be alone in my little place…” [PHR 11110329]* |
| **SELF-ESTEEM AND SELF-ACTUALIZATION** | | | | | | | | |
| **Personal**  **mastery** | | Met needs | | | | *“If you want to get off the streets, if you have the sincere desire to do it, then this is where to do it.”*  *[ESU 11190490]*  *“It’s time to wake up and buckle down to my little minimum wage job; maybe return to school…” [ESU 11190490]*  *It`s me who took the steps. People here helped, but I was the one who went after everything that has been put in place. [ESU 111172750]* | *“I have everything I need in life… It’s me who doesn`t work on myself.” [THR 11131352]*  *“It’s me. It’s my attitude. They offer me services, housing, but nobody can take my place… I’m my best friend, and my worst enemy, depending… Nobody forces me to drink, or sniff, or smoke. I’m the one who messed up my life, so only I can climb back up.” [THR 11121289]* | *“The chance to be here allowed me to start over. And that gave me confidence in myself.” [PHR 11199296]*  *“Myself: I’ve learned to take myself one day at a time.” [PHR 11173302]*  *“Independence, that’s what I enjoy most. It’s my own responsibility, and then rely on that and don`t count on anybody else. I’m really proud of that….” [PHR 11130312]* |
|  |  | Unmet needs | | | | *“…I’m an artist. I wanna use my talent to say to the world that it’s possible to develop your talent, and pass it on…“*  *[ESU 11117250]* | *“After an exhausting year, I was hoping to go there (TH), have my own room, rest up, and plan what to do. Instead, it was two months of pure hell. I had to take medication because I couldn’t sleep… If you knew what was going on there.”*  *[THR 11114240]*  *“I would like to be able to breathe a bit, to live. Because I don’t think I am living well. It’s hard.” [THR 11114441]*  *“I know very well that it’s up to me to take care of myself; but I have a tendency to ask others to help me all the time, which isn`t good. It’s better to rely on oneself.” [THR 11140381]*  *“I lost my daycare, but I was hoping to build another business; that would have realized my dreams.” [THR 11153461]* | *“The housing did nothing to help me make positive changes; I was no closer to being able to work, get an apartment or have a social life. My life was a perpetual starting over again.” [PHR 111527849]*  *“I want to be more empathetic, and show others that this can help society… Because people don`t have enough concern for others. You hear very often, ‘this isn`t my problem.” [PHR 11164673]* |
